# Four decades of screening and brief alcohol intervention research: the peg and the hole

**DOI:** 10.1093/eurpub/ckac152

**Published:** 2022-11-09

**Authors:** Per Nilsen, Sven Andréasson

**Affiliations:** Department of Health, Medicine and Caring Sciences, Linköping University, Linköping, Sweden; Centre for Dependency Disorders, Stockholm Health Care Services, Stockholm County Council, Stockholm, Sweden; Department of Global Public Health, Karolinska Institutet, Stockholm, Sweden

The World Health Organization (WHO) has pursued a population-based approach to address alcohol problems since the late 1970s. The WHO initiative produced the Alcohol Use Disorders Identification Test screening tool and facilitated the development of a screening and brief intervention (SBI) strategy for non-treatment-seeking, non-alcohol-dependent, hazardous and harmful drinkers. SBI is intended as secondary prevention of alcohol-related problems in general healthcare settings. SBI implies screening every patient for problematic alcohol use and subsequently intervening with patients identified as drinking above recommended levels.

A large body of SBI research has accumulated over four decades. The scientific literature provides a fairly solid evidence base for the efficacy and effectiveness of SBI at reducing hazardous and harmful alcohol consumption. Still, many studies have documented that SBI has had minimal impact on routine healthcare practice. Studies of SBI implementation projects in numerous countries have documented limited use of SBI.

Factors that affect the implementation of SBI have been investigated since the late 1970s. Shaw *et al.*^1^ documented poor role legitimacy among physicians in terms of uncertainty as to whether alcohol problems really are their responsibility. Heavy workload and pressed working conditions for health professionals in primary healthcare provide another important explanation. Insufficient skills and knowledge as well as inadequate resources to address alcohol have also been documented. Further SBI barriers include system- and organization-level factors such as insufficient leadership and poor management support for alcohol prevention activity. Lack of financial incentives has also been identified as a barrier.

Given the many barriers to implementing and delivering SBI, one may ask whether this strategy really is feasible in routine practice. SBI entails screening of every patient for problematic alcohol use and subsequently intervening with patients identified as drinking at hazardous or harmful levels. However, general screening to detect patients with problem drinking is usually perceived as unrealistic by health professionals because it is an additional time-consuming task that is not considered a natural part of the clinical work. Studies have reported negative effects of general screening on the physician–patient relationship.

After screening, health professionals should provide a brief intervention with problem-drinking patients. The median duration of brief interventions in a widely cited Cochrane review by Kaner *et al.*^2^ was more than 25 min. This can be compared with recent studies carried out in Sweden, Norway, the Netherlands and England, which have shown that the vast majority of alcohol conversations in routine healthcare are shorter than 1 min. Thus, research-based SBI does not seem congruent with the realities of everyday practice in healthcare.

Alcohol consumption is potentially relevant for many and diverse symptoms, health problems and disorders. In populations where a large majority consume alcohol, such as in Europe, it is clinically highly relevant to address alcohol for common health problems that may be attributable to or complicated by alcohol, e.g. hypertension, depression, psoriasis, in particular for older adults. Identifying clinical signs or health problems that might be associated with problematic alcohol use is referred to as targeted screening or pragmatic case finding.

A study by Reinholdz *et al.*^3^ compared targeted screening with research-based SBI, i.e. general screening and provision of brief intervention and found that SBI identified 70% of all hazardous drinkers compared with 38% with the former approach. This indicates that targeted screening may fail to identify substantial proportions of problem drinkers, yet it could be argued that, if SBI is hardly carried out at all, targeted screening may still confer a considerable public health benefit.

Research should investigate the impact of targeted screening on alcohol consumption and associated problems. Studies are needed to investigate the efficiency in terms of identifying problem drinking and the extent to which health professionals are knowledgeable in recognizing specific clinical signs where alcohol may be relevant, both causally and as a complicating factor.

In conclusion, we believe targeted screening is worthy of further investigation. Alcohol prevention research must expand beyond the current parameters of SBI research and adapt to the realities of healthcare practice. We agree with Kaner who noted in 2010^4^ (p. 589) that this research ‘is in vain if practitioners are unwilling or unable to use these interventions with their patients’. Conducting more SBI research with the ambition that the findings will contribute to increased implementation seems rather like trying to fit a square peg (alcohol prevention based on SBI) into a round hole (everyday healthcare practice). We believe it would be more productive to conduct research based on the recognition that the peg has to be adapted to fit the hole.

*Conflicts of interest*: None declared.
